# A simple and fast heuristic for protein structure comparison

**DOI:** 10.1186/1471-2105-9-161

**Published:** 2008-03-25

**Authors:** David A Pelta, Juan R González, Marcos Moreno Vega

**Affiliations:** 1 Models of Decision and Optimization Research Group, Dept. of Computer Science and Artificial Intelligence, University of Granada, Spain; 2 Department of Statistics, Operations Research and Computation (DEIOC), University of La Laguna, Spain

## Abstract

**Background:**

Protein structure comparison is a key problem in bioinformatics. There exist several methods for doing protein comparison, being the solution of the Maximum Contact Map Overlap problem (MAX-CMO) one of the alternatives available. Although this problem may be solved using exact algorithms, researchers require approximate algorithms that obtain good quality solutions using less computational resources than the formers.

**Results:**

We propose a variable neighborhood search metaheuristic for solving MAX-CMO. We analyze this strategy in two aspects: 1) from an optimization point of view the strategy is tested on two different datasets, obtaining an error of 3.5%(over 2702 pairs) and 1.7% (over 161 pairs) with respect to optimal values; thus leading to high accurate solutions in a simpler and less expensive way than exact algorithms; 2) in terms of protein structure classification, we conduct experiments on three datasets and show that is feasible to detect structural similarities at SCOP's family and CATH's architecture levels using normalized overlap values. Some limitations and the role of normalization are outlined for doing classification at SCOP's fold level.

**Conclusion:**

We designed, implemented and tested.a new tool for solving MAX-CMO, based on a well-known metaheuristic technique. The good balance between solution's quality and computational effort makes it a valuable tool. Moreover, to the best of our knowledge, this is the first time the MAX-CMO measure is tested at SCOP's fold and CATH's architecture levels with encouraging results.

Software is available for download at .

## Background

The comparison of the 3D structures of protein molecules is a challenging problem. The search for effective solution techniques is required because such tools aid scientists in the development of procedures for drug design, in the identification of new types of protein architecture, in the organization of the known universe of protein structures and could assist in the discovery of unexpected evolutionary and functional inter-relations among them.

Moreover, good protein structures comparison techniques could be also used in the evaluation of *ab-initio, threading or homology modeling *structure predictions. It is claimed that the comparison of proteins' structures, and subsequent classification (according to similarity) is a fundamental aspect of today's research in important fields of modern Structural Genomics and Proteomics [1-3].

Several types of strategies and methodologies are applied for protein structure comparison and it is out of the scope of this work to perform an exhaustive review. As a showcase, we may cite the use of dynamic programming [4], comparisons of distance matrices [5], graph theory [6], geometrical hashing [7], principle component correlation analysis [8], local and global alignment [9], consensus shapes [10], consensus structures [11], Kolmogorov complexity [12], Fuzzy Contact Map Overlap [13], and comparing proteins as paths in 3D [14]. The interested reader in the field of structural bioinformatics may refer to [3,15,16] for updated information.

The Maximum Contact Map Overlap problem (MAX-CMO) is a mathematical model that allows to compare the similarity of two protein structures. This model represents each protein as a contact map where spatially close elements of interest are indicated in a matrix. Then, the objective is to construct an alignment that maximizes certain cost. An alignment indicates a correspondence between the elements (amino acid residues or atoms) of both proteins.

In these last years, exact algorithms for solving MAX-CMO have been developed. Among them, we should cite the initial work of [17] and then extended in [18-20]. More recently, [21,22] presented another strategy for optimally solving MAX-CMO. However, we may find several reasons to justify the application of approximate algorithms for MAX-CMO:

• the problem of maximizing the overlap between two contact maps is NP-hard [17,23], existing particular problem instances, i.e. particular pairs of contact maps, where the exact algorithms may fail to return a solution in reasonable time.

• exact algorithms are expensive and hard to code. For example, they may involve (as in [20]) the usage of a local search strategy or even a genetic algorithm for obtaining lower bounds (with their corresponding parameter setting), or a linear programming solver for obtaining upper bounds. Moreover, if a running time limit is established, they may finish without any solution at all.

• the availability of exact methods is limited. To the best of our knowledge, just the algorithm presented in [21] is available trough Internet [24], although there is a limitation on the size of submitted problems (no more than 100 residues) and the CPU time given for solving it (a maximum of 10 minutes).

• MAX-CMO aims to maximize a purely geometrical relation between graphs so a set of suboptimal solutions may also provide insights in terms of the biological meaning of the alignment.

• due to potential errors in the 3D coordinates determination, we may argue against the usefulness of having exact solutions for protein pairs coming from (maybe) erroneous contact maps. As stated in [25], the experimental errors in the determination of the atomic Cartesian coordinates by X-Ray Crystallography or NMR may range from 0.01 to 1.27Å which is close to the value of some covalent bonds.

In this work, we pursue two objectives: firstly, we propose a Variable Neighborhood Search (VNS) strategy for solving MAX-CMO and we show that this strategy allows to obtain near optimal results using reduced computational resources and time.

Secondly, the role of MAX-CMO for doing clustering and classification has only been done at the SCOP's family level (in the so called "Skolnick's dataset) and we propose to assess if the (normalized) overlap values returned by our strategy offers a proper ranking of structural similarity at other different structural levels.

## Results

The validation of the proposed method is done through two different computational experiments: we compare the VNS approach against the exact algorithm from Xie and Sahinidis [21] in pure optimization terms; then we verify if our VNS is able to obtain a proper ranking among a set of proteins at different levels of structural similarity.

### 0.1 Is VNS beneficial from an optimization point of view?

In the first computational experiment we compare our VNS implementation against the results from [21]. As test bed for comparison, we use two datasets described in [26] (see Table 1 for details): a) Skolnick, with 40 proteins and 161 optimally solved pairs, and b) Lancia, with 269 proteins and 2702 optimally solved pairs.

**Table 1:** Datasets' information. "Pairs" stands for the number of pairwise comparisons performed. The values for "Contacts" corresponds to contact maps at 7Å.

			Residues			Contacts		
			
Dataset	Proteins	Pairs	Min.	Avg.	Max.	Min.	Avg.	Max.
Lancia	269	2702	44	57,07	68	33	95,91	137
Skolnick	40	161	97	158,23	255	265	470,93	815
Fischer	68	4624	56	211,16	581	147	636,66	1952
Nh3D	806	58838	33	150,33	759	74	432,83	2438

We use the contact map data files provided in [24] for a fair comparison and reproducibility purposes. The maps are based on *C*_*α *_and the optimal overlap values were kindly provided by the authors of Ref. [21].

We limited the experiment is limited to those cases (protein pairs) where the exact algorithm was able to find the optimum within the time of ten days [21].

Experiments on [21], were conducted on three workstations with a 3.0 Ghz CPU and 1.0 Gb of RAM each while our experiments were run on just one workstation with a 2.2 Ghz CPU (AMD Athlon 64 3500+) and 1Gb of RAM. Xie and Sahinidis have recently improved the results from [21] in terms of computing resources needed [22].

We define three versions of our Multistart VNS (MSVNS), corresponding to different parameter settings for one of the neighborhood structures (see details in the Methods section):

• MSVNS1: One neighborhood of type *neighborhoodMove *and 3 neighborhoods of type *neighborhoodAdd*, having window sizes of 5%, 10% and 15% respectively.

• MSVNS2: One neighborhood of type *neighborhoodMove *and 3 neighborhoods of type *neighborhoodAdd *having window sizes of 10%, 20% and 30% respectively.

• MSVNS3: One neighborhood of type *neighborhoodMove *and 3 neighborhoods of type *neighborhoodAdd *having window sizes of 10%, 30% and 50% respectively.

The strategy has a parameter that controls the number of internal "restarts": i.e. when no improvement can be done from the incumbent solution, the search is restarted from a new randomly generated one. This value is fixed in 150. At the end of the execution, we measure the *error*(%) with respect to the optimum value. The results are shown in Tables 2 and 3.

**Table 2:** Results over 2702 pairs from Lancia's dataset. The error is measured with respect to the optimum value.

			Error (%)		
			
	Version	N	Avg.	SD	Median
Total	MSVNS1	2702 (100%)	5,8765	7,12280	1,9049
	MSVNS2	2702 (100%)	3,9959	5,60979	0,0000
	MSVNS3	2702 (100%)	3,5671	5,21332	0,0000

Optimally Solved	MSVNS1	1259 (46,60%)	0,0000	0,00000	0,0000
	MSVNS2	1522 (56,33%)	0,0000	0,00000	0,0000
	MSVNS3	1577 (58,37%)	0,0000	0,00000	0,0000

Non-Optimally Solved	MSVNS1	1443 (53,40%)	11,0037	6,21068	11,1111
	MSVNS2	1180 (43,67%)	9,1499	4,98958	9,0909
	MSVNS3	1125 (41,63%)	8,5674	4,73640	8,3333

**Table 3:** Results over 161 pair from Skolnick's dataset. The error is measured with respect to the optimum value.

			Error(%)		
			
	Version	N	Avg	SD	Median
Total	MSVNS1	161 (100%)	7,3950	7,44111	5,5556
	MSVNS2	161 (100%)	1,8235	2,50117	0,7375
	MSVNS3	161 (100%)	1,6744	2,39488	0,4399

Optimally Solved	MSVNS1	42 (26,09%)	0,0000	0,00000	0,0000
	MSVNS2	62 (38,51%)	0,0000	0,00000	0,0000
	MSVNS3	68 (42,24%)	0,0000	0,00000	0,0000

Non-Optimally Solved	MSVNS1	119 (73,91%)	10,005	6,98169	9,5092
	MSVNS2	99 (61,49%)	2,9655	2,60624	2,2124
	MSVNS3	93 (57,76%)	2,8987	2,52732	2,3910

The main thing to notice from both Tables is that as the windows sizes increases, the average error decreases. The best alternative is MSVNS3 with windows sizes of 10–30–50 leading to an average error below 3.6% for Lancia's dataset with 2702 pairs, and below 1.7% for the Skolnick's one. As the median values are much lower than the average, Tables also show the number of pairs that were optimally solved and those where the optimum was not reached. For Lancia's dataset, up to 60% of the pairs can be optimally solved, while in Skolnick, the percentage of optimum was around 40%. Again, the percentages of non-solved pairs diminishes as the windows' sizes increases.

**Figure 1:**
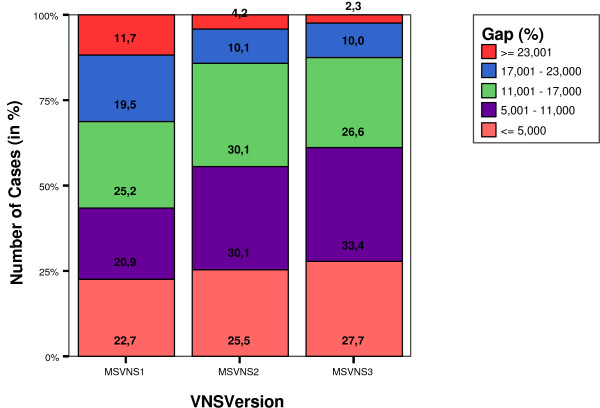
Distribution of gaps (error) from exacts values (in %) in the set of non optimally solved solutions for Lancia's dataset.

**Figure 2:**
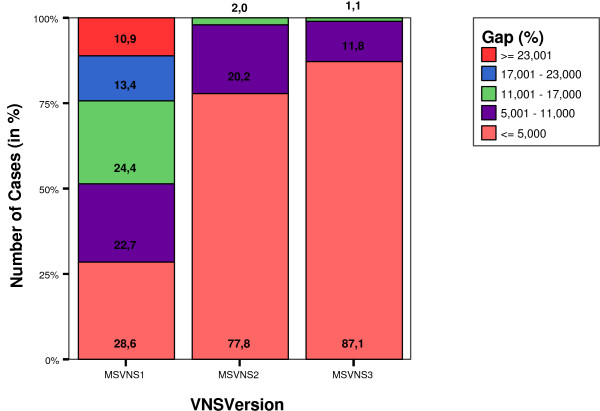
Distribution of gaps (error) from exacts values (in %) in the set of non optimally solved solutions for Skolnick's dataset.

It is also interesting to analyze the subset of pairs that were not optimally solved. Figure 1 shows the distribution of such pairs on the Lancia's dataset over five different ranges of percentage of error, for each of the three VNS versions. Figure 2 shows the same graph for Skolnick's dataset.

It can be seen that the quality of the results increases as the VNS version goes from MSVNS1 to MSVNS3 where both, the number of non-optimally solved solutions and the corresponding error are the smallest. The error is below 11% for more than half of the pairs in all cases for Lancia's dateset. When using MSVNS3 the errors get as low as having 87% of the non-optimal pairs solved with less than 17% of error. For Skolnick, MSVNS3 obtains an error lower than 5% for the 87% of non optimally solved pairs.

Regards to computational effort needed to achieve these results, Table 4 reflects the total wall clock time for every variant of MSVNS. The bigger the window sizes, the longer the times. This is because as these sizes increase, the number of potential pairings becomes larger, leading to an expected increase of execution time. However, we consider the tradeoff between solutions quality and computational effort as highly reasonable.

**Table 4:** Wall clock time required for each variant of VNS to solve all the pairs from each dataset. The number of pairs was 2702 for Lancia's dataset and 161 for Skolnick's one.

	Time	
	
Version	Lancia	Skolnick
MSVNS1	4 hs. 12 m.	3 hs. 11 m.
MSVNS2	4 hs. 43 m.	4 hs. 48 m.
MSVNS3	5 hs. 7 m.	5 hs. 44 m.

These results confirm that the proposed strategy is a useful tool for solving near optimality MAX-CMO for almost all the evaluated protein pairs.

### 0.2 Is VNS able to rank properly protein similarity ?

Although the analysis from an optimization point of view is relevant, it is also interesting to check the quality of VNS as a protein structure classifier. In other words, we want to assess if it is really necessary to solve MAX-CMO exactly to perform structure classification.

Moreover, overlap values are not adequate *per se *for classification purposes because such values depend on the size of the proteins being compared. Indeed a normalization scheme should be applied and we illustrate that this may play a crucial role in protein classification.

There is no general agreement on how to do normalization, but at least, three alternatives are available.

1. *norm*1(*P*_*i*_, *P*_*j*_) = *overlap*(*P*_*i*_, *P*_*j*_)*/min*(*contacts P*_*i*_, *contacts P*_*j*_)

2. *norm*2(*P*_*i*_, *P*_*j*_) = 2 * overlap(*P*_*i*_, *P*_*j*_)/(*contacts P*_*i *_+ *contacts P*_*j*_)

3. $norm3(P_{i},P_{j})=\{\begin{array}{ll} 0 & \text{if the contacts difference is greater than }75\%\\ norm1(P_{i},P_{j}) & \text{otherwise} \end{array}$

First and second alternatives were proposed in [3] and [21] respectively. Here, we propose the third alternative to avoid the comparison of two structures whit completely different sizes.

We perform three computational experiments to analyze our proposal. Firstly, we made an all against all comparison in Skolnick's dataset to check wether a clustering can discriminate among 5 SCOP families. Secondly, we test the performance of the strategy to detect similarity at SCOP's fold level, using Fischer's dataset [27]. For this experiments, comparison with DaliLite is also performed. Finally, we made a set of queries over the NH3D database [28] to evaluate the capability of a nearest neighbor classification to detect similarity at CATH's architecture level. Comparisons are then made against DaliLite and MatAlign.

#### 0.2.1 Experiments with Skolnick's dataset

For this experiment we use again Skolnick's dataset from [21,22], which includes a "cluster" label for every protein corresponding to different families in SCOP database [29]. We perform an all against all comparison among the proteins using the best (MSVNS3) and worst (MSVNS1) versions of our strategy (from an optimization sense). Then we constructed a similarity matrix for each MSVNS configuration using the overlap values normalized with *Norm1, Norm2, Norm3*. Finally, we apply single and average linkage hierarchical clustering as implemented in R software package [30] with the final objective of evaluating if the original grouping can be recovered from the overlap values or not.

**Figure 3:**
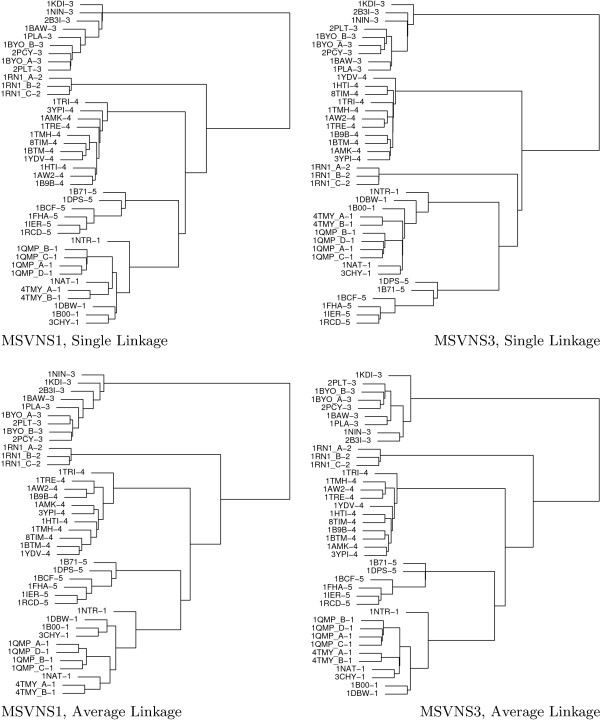
Hierarchical Clustering based on the normalized overlap values (using *Norm*1) among proteins in Skolnick's dataset. The upper dendrograms (a, b) correspond to single linkage clustering and the lower ones (c, d) to average linkage clustering. Dendrograms on the left (a, c) are for MSVNS1 results while dendrograms on the right (c, d) correspond to MSVNS3 results.

Both, MSVNS1 and MSVNS3, are able to perfectly recover the original grouping independently of the normalization and clustering algorithms. Figure 3 shows particular examples, where single and average linkage clustering are applied over the similarity matrix normalized with *Norm1*. For visualization purposes, the class number is displayed at the right of the protein name.

The study performed in this dataset shows that our strategy can replicate the results obtained using exact methods but with less computational effort and a simple strategy. Moreover, this experiment confirms that correct classification may be performed using non-exact Max-CMO values. Both elements are important results *per se*.

#### 0.2.2 Experiments on Fischer's dataset

We perform a second experiment using Fischer's dataset (described in Table II from [27]) composed by 68 proteins and comprising several classes and folds. Table 1 provides information about protein sizes.

An all-against-all comparison was performed using MSVNS2 and DaliLite [31] with default parameters. In this case, the objective is to analyze the ability of the methods to recognize structural similarities at (SCOP) fold level.

**Figure 4:**
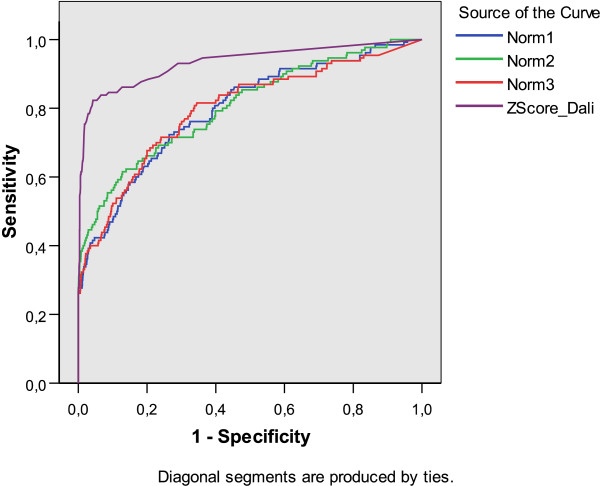
ROC curves for every measure on Fischer's dataset at fold level.

**Figure 5:**
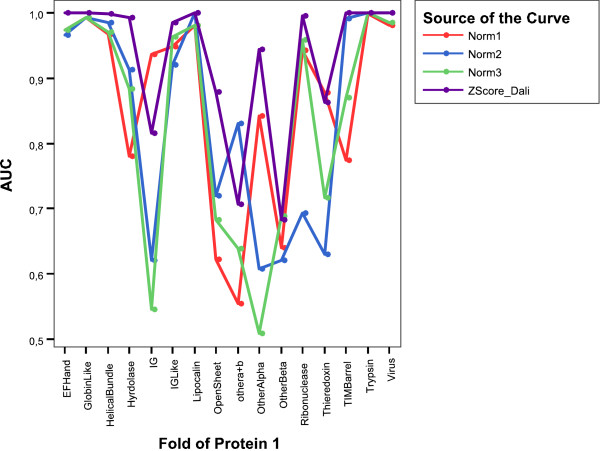
AUC values for every measure on Fischer's dataset for every type of fold.

Figure 4 shows the global ROC curves for fold level, while Table 5 shows the corresponding area under curve (AUC), calculated with SPSS 14.03©. Notably DaliLite achieved the highest AUC's values. However, when we discriminate AUC's values in terms of the fold, as shown in Fig. 5, two notorious elements should be highlighted. First, we found that for some folds DaliLite is not the best, and second, each normalization is able to detect certain types of folds, while failing in others. For example, the IG fold is best detected with *Norm*1, while this measure gives the lowest AUC value for TIM-barrel fold.

**Table 5:** Area Under the Curve (AUC) for each measure over Fischer's dataset at fold level. An all against all comparison is performed among the 68 proteins in the dataset.

				Asymptotic 95% Conf. Interval	
				
Variable	Area	Std. Error^(*a*)^	Asymptotic Sig.^(*b*)^	Lower Bound	Upper Bound
*norm*1	0,795	0,016	0.000	0,765	0,826
*norm*2	0,805	0,016	0.000	0,774	0,836
*norm*3	0,797	0,016	0.000	0,765	0,830
*DaliLiteZScore*	0,933	0,011	0.000	0,912	0,954

^(*a*) ^Under the nonparametric assumption

^(*b*) ^Null hypothesis: true area = 0.5

**Figure 6:**
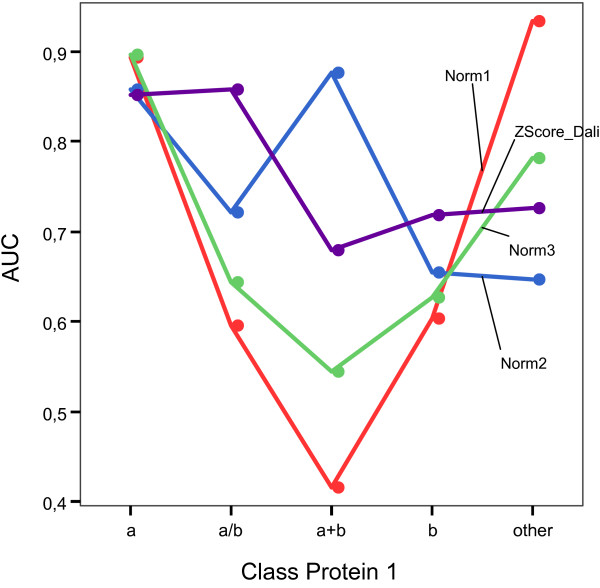
AUC values for every measure on Fischer's dataset for every class.

Moreover, when we discriminate the results in terms of the class of the first protein in the pair, we obtain again some interesting results that are shown in Fig. 6. Table 6 shows the corresponding area under curve (AUC). DaliLite obtained the highest AUC value in just two (a/b, b) out of 5 classes. In the other cases, the highest value is obtained by some of the normalizations based on the overlap returned by MSVNS. For a total of 68 × 68 = 4624 pairwise comparison, DaliLite detected no similarity for 2800 pairs (60.5%). If we consider those pairs with z-score < 1, then the value grew to 3844 (83.1%).

**Table 6:** Area Under the Curve (AUC) for each measure over Fischer's dataset at class level. An all against all comparison is performed among the 68 proteins in the dataset.

				Asymptotic 95% Conf. Interval	
				
Variable	Area	Std. Error^(*a*)^	Asymptotic Sig.^(*b*)^	Lower Bound	Upper Bound
*norm*1	0,601	0,010	0.000	0,582	0,621
*norm*2	0,678	0,009	0.000	0,661	0,696
*norm*3	0,637	0,009	0.000	0,619	0,656
*DaliLiteZScore*	0,772	0,009	0.000	0,755	0,789

^(*a*) ^Under the nonparametric assumption

^(*b*) ^Null hypothesis: true area = 0.5

#### 0.2.3 Experiments on Nh3D database

The last test is done using the Nh3D v3.0 dataset [28] of structurally dissimilar proteins. This dataset has been compiled by selecting well resolved representatives from the Topology level of CATH database. These have been been pruned to remove domains that may contain homologous elements, internal duplications and regions with high B-Factor.

Our aim is to check if MSVNS2 can properly classify structures at CATH's architecture level. The database has 806 topology representatives belonging to 40 architectures. Table 1 provides information about protein sizes.

For each architecture (comprising at least 10 topologies) we select the smallest, biggest and average structure in terms of residues and number of contacts, plus another one randomly selected. After removing duplicates, we obtained a set of 73 structures that constitutes the query set [see Additional File 1]. Each query is then compared against every structure in the database. Comparisons are also performed with DaliLite [31] and a recently proposed method based as well on distance matrices, MatAlign, claimed to be better than DaliLite and CE [32] in certain cases [33]. For the former, we use the z-score as similarity measure, while the raw score is used for the later. For MSVNS2, we made the analysis using the three normalization schemes proposed above. The number of internal restarts for MSVNS2 was fixed to 10 to constraint the execution time up the computation.

**Figure 7:**
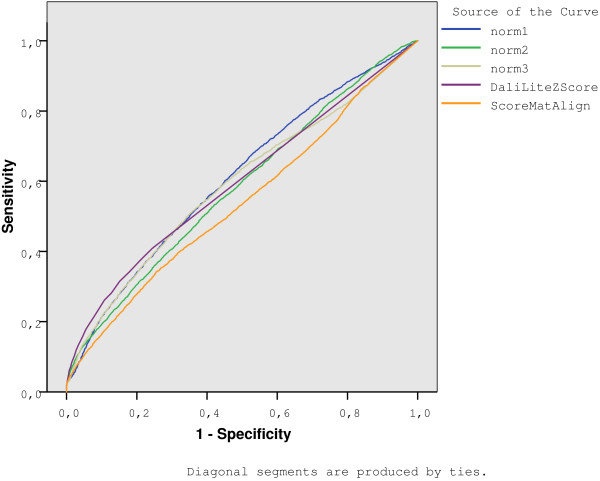
ROC curves for every measure on NH3D dataset at CATH's architecture level.

Figure 7 displays the ROC curve for every method while Table 7 reports the corresponding "area under curve" (AUC) values. Again, we note that normalization is a key factor and different conclusions may be obtained. When normalization is *Norm1*, the AUC value is higher than that of DaliLite. Other alternatives obtain lower values. MatAlign obtains the lowest result. It is important to recall that *Norm*1, *Norm*2, *Norm*3 are based on the overlap value returned by our strategy.

**Table 7:** Area Under the Curve (AUC) for each measure over NH3D database. The experiment consisted on 73 queries over 806 domains. The analysis is performed at CATH's family level.

				Asymptotic 95% Conf. Interval	
				
Variable	Area	Std. Error^(*a*)^	Asymptotic Sig.^(*b*)^	Lower Bound	Upper Bound
*norm*1	0.608	0.005	0.000	0.599	0.618
*norm*2	0.582	0.005	0.000	0.572	0.591
*norm*3	0.591	0.005	0.000	0.581	0.601
*DaliLiteZScore*	0.596	0.005	0.000	0.586	0.607
*ScoreMatAlign*	0.538	0.005	0.000	0.528	0.548

^(*a*) ^Under the nonparametric assumption

^(*b*) ^Null hypothesis: true area = 0.5

**Figure 8:**
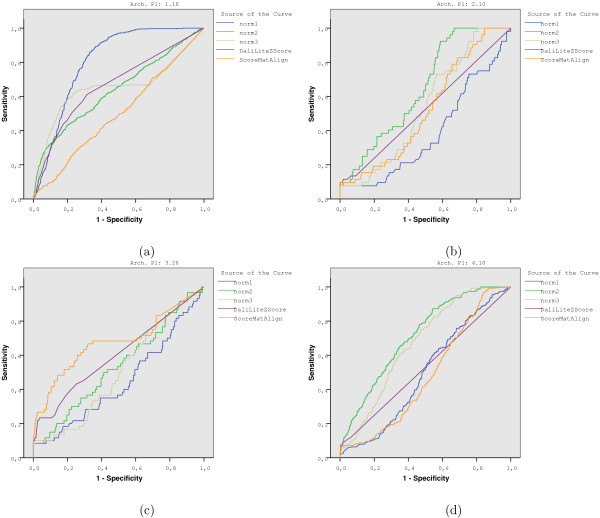
Examples of ROC curves to show how dependent the strategies are on the architecture type of the query.

If we trace different curves in terms of the architecture of the query, we may find several interesting behaviors. Some examples of ROC curves are displayed in Figure 8 where clear differences arise among methods as a function of the query's architecture.

**Figure 9:**
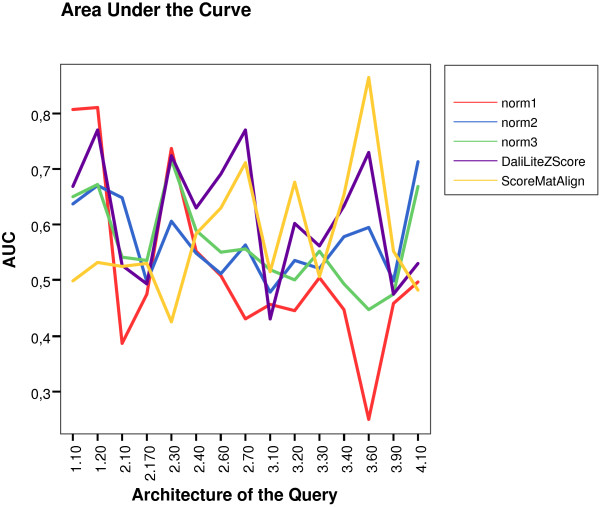
AUC values for every measure and type of query's architecture on NH3D dataset. It is clear that none of the methods stays on top of the other ones for all the architecture's types.

Figure 9 displays the corresponding AUC values for every architecture, excluding those where all methods achieved AUC = 1. If we assume AUC values as a measure of similarity detection "hardness" then, it is clear that this concept is different for every scoring scheme. From this Figure, we note that no single algorithm outperforms the others for every possible query's architecture.

It should be noted that, from a total of 73 × 806 = 58838 pairwise comparisons, DaliLite detected no similarity for 43833 pairs (74.5%), leading to several false negatives. As an example, for two out of seven queries belonging to architecture 4.10, DaliLite failed to return these queries as the most similar structures in the database. When the process is repeated with MSVNS, the query itself is given the highest value of similarity in the seven cases.

## Discussion and Conclusion

In this work we tested a straight and simple implementation of VNS for the MAX-CMO problem which obtains encouraging results.

¿From an optimization point of view, MSVNS obtained overlap values that were very close to the optimal ones, using a simpler strategy and less computational effort than exact algorithms.

We can mention at least, three ways to obtain further improvements to our method: a) by trying more specialized neighborhood structures, b) by better tuning the parameters' values chosen c) by starting the search from heuristically generated solutions. We also plan to add a preprocessing step to avoid making comparisons between structures that are very different, as DaliLite does. Moreover, due to its speed and simplicity, VNS may be also considered for obtaining lower bounds in the context of exact algorithms. An important element in several bioinformatics problems is the relation between the optimum value of the objective function and the biological relevance of the corresponding solution. In protein structure comparison we should remember that we are dealing with a mathematical model that captures some aspects of the biological problem, being possible to measure protein structure similarity in several ways. For example, up to 37 measures are reviewed in [34]. Moreover, besides obtaining the highest overlap values, it is also critical to develop strategies able to obtain a proper similarity ranking of proteins.

Our experiments showed that in terms of SCOP's family and CATH's architecture levels, the (normalized) overlap values seemed to be good enough to capture the similarity. At the level of SCOP's fold, several points should be consider. Although DaliLite outperformed MSVNS2, it does not imply that our method did badly. More research should be done, specially in the area of normalization, because, as we mentioned the use of different normalization schemes may lead to stronger or weaker strengths to detect particular kinds of folds. We should also recall that all of the experiments were done using contact maps with a fixed threshold and it may be the case that for detecting similarity at fold level, a different value would be needed. Wether the performance of DaliLite for detecting similarity at fold level can be achieved or not with a strategy based on the contact maps model remains open.

Just to conclude, we should mention that the method was accepted to be incorporated on the ProCKSI-Server [35]. ProCKSI is a decision support system for Protein (Structure) Comparison, Knowledge, Similarity and Information that computes structure similarities using information theory measures. ProCKSI links to a variety of other sources and uses additional methods to rectify and augment its similarity findings. Our MSVNS was chosen as the method to solve MAX-CMO due to its speed and accuracy.

## Methods

### 0.3 The Maximum Contact Map Overlap Problem

**Figure 10:**
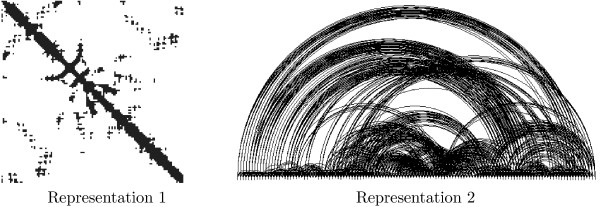
Two contact map representations: as binary matrix (left) and as a graph (right).

The Maximum Contact Map Overlap problem (MAX-CMO) is a mathematical model that allows to compare the similarity of two protein structures. Under this model, each protein is represented as a contact map (a binary matrix) where two residues are said to be in contact if their Euclidean distance in 3D is below a certain threshold ℜ. Figure 10 shows two alternative representations for a contact map.

A solution for two contact maps is an alignment of residues (i.e a correspondence between residues in the first contact map to residues on the second one). Aligned or paired residues are considered to be equivalent. The pairings are not allowed to cross: if there exists a pairing *i *↔ *j *that aligns residues *i *∈ *P*_1_, *j *∈ *P*_2_, then it is not allowed that any other pairing of the form *a *↔ *b, a *≥ *i*, *b *≤ *j *exists at the same time.

In MAX-CMO, the value of an alignment between two proteins is given by the number of cycles of length four. This number is called the overlap of the contact maps and the goal is to maximize this value, i.e. the larger this value, the more similar the two proteins.

**Figure 11:**
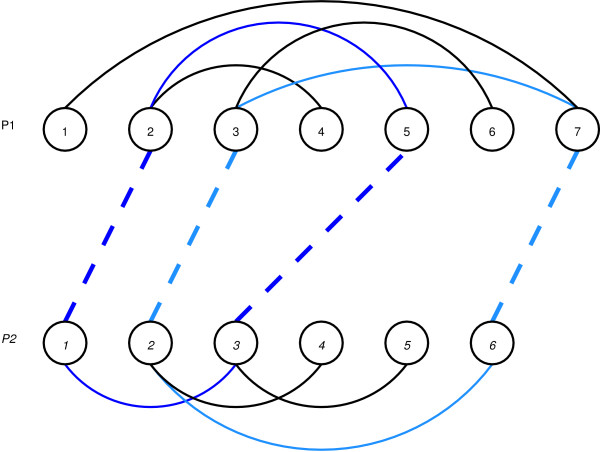
An example of an alignment between two schematic proteins. The overlap value is 2 because two cycles of length four arise. First cycle is composed by the arcs (2 ∈ *P*_1_, 5 ∈ *P*_1_), (5 ∈ *P*_1_, 3 ∈ *P*_2_), (3 ∈ *P*_2_, 1 ∈ *P*_2_) and (1 ∈ *P*_2_, 2 ∈ *P*_1_). The second cycle has the following four arcs: (3 ∈ *P*_1_, 7 ∈ *P*_1_), (7 ∈ *P*_1_, 6 ∈ *P*_2_), (6 ∈ *P*_2_, 2 ∈ *P*_2_) and (2 ∈ *P*_2_, 3 ∈ *P*_1_).

An example appears in Fig. 11 where two contact maps are shown. Residues 2, 3, 5 and 7 in the upper protein (*P*_1_) are paired with residues *1, 2, 3 *and *6 *in the lower one (*P*_2_). The alignment is represented by dotted lines while the protein' contacts are shown with solid ones.

This particular alignment produces two cycles of length four. First cycle is composed by the arcs (2 ∈ *P*_1_, 5 ∈ *P*_1_), (5 ∈ *P*_1_, 3 ∈ *P*_2_), (3 ∈ *P*_2_, 1 ∈ *P*_2_) and (1 ∈ *P*_2_, 2 ∈ *P*_1_). The second cycle has the following four arcs: (3 ∈ *P*_1_, 7 ∈ *P*_1_), (7 ∈ *P*_1_, 6 ∈ *P*_2_), (6 ∈ *P*_2_, 2 ∈ *P*_2_) and (2 ∈ *P*_2_, 3 ∈ *P*_1_).

### 0.4 MultiStart VNS metaheuristic

Variable Neighborhood Search (VNS) metaheuristic was presented in [36,37]. It is essentially a local search method which includes dynamic changes in the neighborhood of the solutions.

VNS for MAX-CMO aims to find good solutions by adding and removing pairings using different strategies. The scheme of our proposal is shown in Algorithm 1.

Algorithm 1 Our MultiStart VNS algorithm

**procedure ***MSV NS*()

**1: for **(*start *= 0; *start *< = *numStarts*; *start*++ **) do**

**2:    Initialization: Select the set of neighborhood structures ***N*_*k*_, **for ***k *= 1, ..., *k*_*max*_, **that will be used in the search; Find an initial solution ***x*; **Choose a stopping condition;**

3:    repeat

**4:       Set ***k *← 1;

5:       repeat

**6:          (a) Shaking: Generate a point ***x' ***at random from the ***k*^*th *^**neighborhood of ***x*(*x' *∈ *N*_*k *_(*x*))**;**

**7:          (b) Local search: Apply some local search method with ***x' ***as initial solution; Denote with ***x" ***the so obtained local optimum;**

**8:          (c) Move or not: If the local optimum ***x" ***is better than the incumbent ***x*, **move there (***x *← *x"***), and continue the search with ***N*_1_(*k *← 1)**; otherwise, set ***k *← *k *+ 1**;**

**9:       until ***k *= *k*_*max*_

**10:       simplify (***x***);**

11:    until stop condition is met

12: end for

A basic VNS algorithm usually follows the pattern shown in lines 2–11 (excluding line 10). Our algorithm extends the basic VNS by incorporating an extra loop (line 1) for restart and a simplification scheme (*simplify *function at line 10).

**Figure 12:**
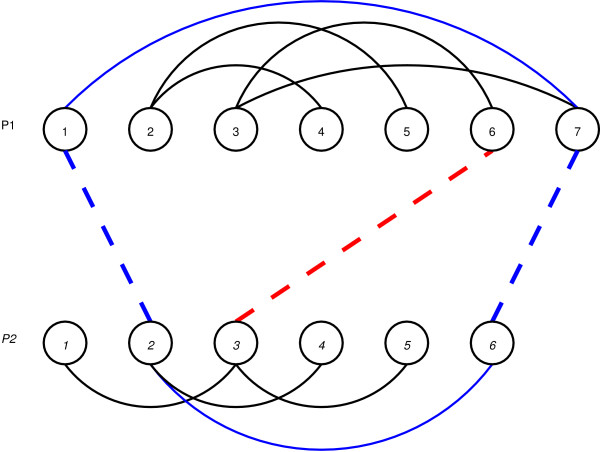
Example of the simplify procedure – Red pairing, from 6 ∈ *P*_1 _to 3 ∈ *P*_2 _will be removed.

This *simplify *function avoids the saturation of solutions and gives more chances to the different neighborhoods used to successfully explore the solution space. It is based on the deletion of useless alignments that do not participate on any cycle of length four. For example in Figure 12 the pairings 2 ↔ 1 and 6 ↔ 7 belong to a cycle (shown in blue), while the pair 3 ↔ 6 will be deleted by the *simplify *function. Two neighboorhood structures are used for the "Shaking" part of the VNS algorithm: a neighborhood that randomly moves a pairing (*neighborhoodMove*); and a neighborhood that adds a random pairing to the alignment (*neighborhoodAdd*).

The function *neighborhoodMove *moves a pairing as follows:

1. it randomly chooses a *pair pairCM1 *↔ *pairCM2*, where *pairCM1 *is the residue on contact map 1 and *pairCM2 *is the residue on contact map 2.

2. Then it finds the nearest paired residues that *pairCM1 *has both to the left (*pairCM1Left*) and to the right (*pairCM1Right*) and the residues in contact map 2 that they are paired to (*pairCM2Left *and *pairCM2Right *respectively).

3. Once these two intervals are determined, the original *pairCM1 *↔ *pairCM2 *pair is replaced by a *pairCM1*' ↔ *pairCM2*' pair where *pairCM1*' ∈ [*pairCM1Left + 1, pairCM1Right - 1*] and *pairCM2*' ∈ [*pairCM2Left + 1, pairCM2Right - 1*].

**Figure 13:**
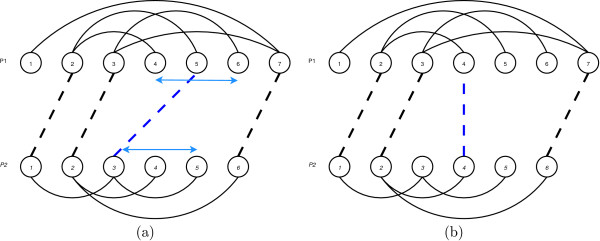
Example of neighborhoodMove procedure. A pairing to move is chosen and a feasibility region is identified (a). An alternative pairing is selected from such region and the original pairing is replaced (b).

The application of this function keeps the feasibility of the solution. An example is shown in Fig. 13 where the pair 3 ↔ 5 can be moved to a pair from any residue from 3 to 5 in the first contact map, and any residue from 4 to 6 in the second contact map. Finally, the 4 ↔ 4 pair is chosen and the original 3 ↔ 5 pair is removed.

The function *neighborhoodAdd *adds a random pairing to the solution, proceeding as follows:

1. It chooses a random, not paired residue (*pairCM1*) from contact map 1.

2. The algorithm finds *pairCM2Left *and *pairCM2Right *in the same way as *neighborhoodMove *does.

3. Instead of just pairing *pairCM1 *with a residue between *pairCM2Left *and *pairCM2Right*, the range of possible pairings is expanded by the size of a *window*. The new pairing will be *pairCM1 *– *pairCM2' *where *pairCM2*' ∈ [*pairCM2Left – window/2, pairCM2Right *+ *window/*2]. Window sizes are expressed as a percentage of the biggest contact map size (i.e a 10% *window *for two contact maps of sizes 80 and 100, results in a window of size 10 (a ten percent of 100, the biggest size)).

4. Since the pairing added can potentially result on an unfeasible solution, we delete all conflicting preexisting pairings. By this way, this neighborhood adds a pairing and may also delete portions of the solution, raising the chances of reconstructing them in a better way. We note that as the *window *parameter gets high, there are more chances of clearing parts of the solution (thus making room for new pairs).

**Figure 14:**
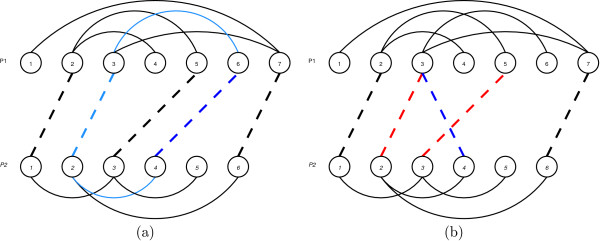
Example of neighborhoodAdd procedure. Residue 4 ∈ *P*_2 _is chosen to be aligned. The pairing may be feasible, as shown in (a) or an unfeasible one (b). In this case, feasibility would be restored by deleting the pairings (3 ∈ *P*_1_, 2 ∈ *P*_2_) and (5 ∈ *P*_1_, 3 ∈ *P*_2_).

Figure 14 shows an example where the random residue chosen to be paired is the fourth from contact map 1. The feasibility restrictions only allow its pairing with residue number 6 from contact map 2, giving the result shown in a). This pairing increments in 1 the overlap value by creating a cycle with the pairs 2 ↔ 3 and 4 ↔ 6. The effect of using the window concept can be seen in b) where it is possible to obtain the pair 4 ↔ 3. Then, feasibility will be restored by removing pairs 2 ↔ 3 and 3 ↔ 5.

These two neighborhood structures are used to define the neighborhoods of the main VNS's loop. In this work, we propose the following configuration:

• k = 1: *neighborhoodMove*.

• k = 2: *neighborhoodAdd *with a small *window *size.

• k = 3: *neighborhoodAdd *with a medium *window *size.

• k = 4: *neighborhoodAdd *with the big *window *size.

So, to keep the basic VNS properties and ideas, the neighborhoods based on *neighborhoodAdd *are always chosen with increasing *window *sizes as *k *increases. For example, the strategy MSVNS3 considers *small *= 10%, *medium *= 20% and *big *= 30%. In this case the search starts *neighborhoodMove*. When VNS cannot improve the overlap value, then the value of *k *is incremented, and the search continues with *neighborhoodAdd *and *window *= 10%. If the failure to improve continued, the process is repeated with *neighborhoodAdd *using *window *= 20%. If necessary, VNS will try with *neighborhoodAdd *using *window *= 30% and when this last neighborhood can not improve the solution, it produces a restart. The local search part of the algorithm uses a different neighborhood structure. It loops from the first to the last residue of contact map 1 and tries to pair it with every feasible residue of contact map 2, making an alignment with the first one that improves the current solution (in a greedy-like fashion).

Finally the stopping criterion for each run of the VNS method is either one hundred iterations or twenty iterations without improvements (whatever comes first).

### 0.5 Time Comparisons

To properly compare the execution time of a set of algorithms, all of them should be ideally compiled and run in the same computational environment. To overcome the lack of source code availability for exact algorithms, we resort to published results. For the case of DaliLite, we did the time comparison after running the algorithm in our local machine.

#### 0.5.1 Times for Exact Methods

In the approach presented in [22], authors have to setup time limits ranging from 4 seconds to 10–30 minutes or even more. Their strategy required one day an a half to optimally solve 1233 pairs from Lancia's dataset. They needed three days to reach 1997 instances and nine, to reach the 2702 instances on a single workstation. Recalling Tables 2 and 4, our worst version MSVNS1 needs approx. 4 hours to solve 2702 pairs, achieving the optimum for 1259 pairs. Unfortunately, execution times for Skolnick's dataset are not provided.

For the approach presented in [20], the code is not available, so we approximate the times for Lancia's dataset looking at the paper:

*We ran our methods on a set of 269 proteins with 64 to 72 residues and 80 to 140 contacts each, using a contact threshold of 5 Å. For the B & C approach we set a maximum time limit of 1 hour or 15 nodes in the search tree per instance the heuristics were applied at every node, and were limited to at most five minutes per node*...

Then, they go on with:

The same 597 pairs were then compared using the LR approach. For each instance we allowed a maximum running time of 1 minute For all instances, the upper bound computed by LR was at least as good as that computed by B & C, however, most of the times bonds were equal. Note that we are not finding the best lagrangian multipliers and hence, in principle, our upper bound may be worse than U1. By using the LR we then compared,..., all 36046 pairs. To speed up the computation, we only explored the root node of the search three and we did not apply the greedy heuristic. Note that running the B&C on all these instances, with a time limit of 1 hour/problem would have taken about four years

Based on these paragraphs we may conclude that a practical use of B& C is not possible and heuristics, like LR, are needed. As such it is possible that a different heuristic finds higher overlap values. The approximate comparison time per pair may be 4.8 seconds (48 hs. (a weekend) divided by 36046 pairs), however the performance of the "greedy heuristic" is not provided.

Although our times are slightly longer than 4.8 sec., we should note that our contact maps had a threshold of 7Å, thus having more contacts per map than those in Lancia's paper and it is not clear how LR execution times would be affected by such increase in the number of contacts.

Moreover, we have a parameter which is linearly related with the speed of the search, namely the number of internal repeats. Time improvement can be easily achieve by setting the number of internal repeats to a low value. Of course, in terms of optimization, the results may degrade, though in terms of classification, it does not produce a significant impact.

#### 0.5.2 Times for DaliLite

To analyze the execution times of DaliLite and MSVNS, we perform a simple experiment. We retrieve the biggest five proteins (1.10.645, 1.20.210, 3.20.70, 3.60.120, 3.90.1300) in terms of contacts availabe in NH3D database; then we perform an all-against-all comparison scheme to filter out those pairs where DaliLite can not detect enough similarity to proceed.

We execute once DaliLite, MSVNS1, MSVNS2 and MSVNS3 on the same machine under the same conditions for every of the remaining eight pairs. Results are reported in Table 8 and they clearly show that our strategy is faster than DaliLite.

**Table 8:** Execution Times for MSVNS and DaliLite. Times correspond to 8 pairwise comparisons among the 5 biggest proteins in NH3D database. Runs were done in the same desktop computer.

Method	Time(sec.)
MSVNS1	357
MSVNS2	508
MSVNS3	613
DaliLite	758

## Authors' contributions

JRG and JMM designed and implemented the VNS strategy based on the ideas of DP's work [13]. DP designed the experiments and JRG ran them. JRG and DP analyzed the results and wrote the paper. All authors have read and approved this final manuscript.

## Supplementary Material

Additional File 1 Query set. This file contains the structures that constitute the query set for the experiment on the Nh3D dataset. For each architecture (comprising at least 10 topologies) we selected the smallest, biggest and average structure in terms of residues and number of contacts, plus another one randomly selected. After removing duplicates, this set of 73 structures was obtained. Click here for file
